# Converting highly productive arable cropland in Europe to grassland: –a poor candidate for carbon sequestration

**DOI:** 10.1038/s41598-017-11083-6

**Published:** 2017-09-05

**Authors:** Paul Gosling, Christopher van der Gast, Gary D. Bending

**Affiliations:** 1 AHDB, Stoneleigh Park, Kenilworth, Warwickshire CV8 2TL UK; 2 School of Healthcare Science, Manchester Metropolitan University, Manchester, M1 5GD UK; 3 School of Life Sciences, University of Warwick, Coventry, CV4 7AL UK

## Abstract

Sequestration of atmospheric CO_2_ as organic carbon by agricultural soils (SOC) is promoted as a climate change mitigation option. IPCC provides guidelines for determining carbon stocks and sequestration potential, incentivising policy changes towards management of farmland for carbon sequestration. However, the basis of the assumption that agricultural soils can sequester significant atmospheric CO_2_ has been questioned. We sought to determine the potential for conversion of arable cropland to grassland to sequester carbon in the short to medium term and potential limiting factors. There were no differences in SOC stocks in the top 30 cm between grassland up to 17 years old and arable cropland at 14 sites across the UK. However, SOC showed different distribution patterns, being concentrated in the top 10 cm under grassland. Soil microbial communities were significantly different between arable and grassland, with higher biomass and lesser dominance by bacteria in grassland soils. A land use conversion experiment showed these changes occurred within one year of land use change. Failure of grassland soils to accumulate SOC was attributed to reduced available soil nitrogen, resulting in low productivity. The implications of these results for carbon sequestration in soils as a climate change mitigation strategy are discussed.

## Introduction

Global climate change, driven by anthropogenic enrichment of the atmosphere with greenhouse gases (CO_2_, CH_4_, N_2_O) is likely to be the key environmental challenge of the twenty-first century. Amongst the range of options suggested to mitigate climate change, manipulation of the global carbon cycle to sequester atmospheric CO_2_ into long-term storage has been extensively promoted. Although industrial methods of direct capture of CO_2_ have been suggested, it is biological capture that has received the most attention^1, 2^. Biospheric carbon sinks are included in international agreements to meet emission reduction targets, with the IPCC suggesting that as much as 180,000 ± 80,000 Tg C could be sequestered by the terrestrial biosphere^3^. Of the terrestrial biosphere sinks, forests and their ability to sequester carbon in trees has received the most attention. However, their potential capacity to capture CO_2_ may have been exaggerated^4, 5^. The other key terrestrial carbon sink is soil. Soils globally contain twice as much carbon (as organic carbon) as the atmosphere and three times as much as the terrestrial biotic pool. As a result, even modest changes in soil organic carbon (SOC) have the potential to significantly influence atmospheric CO_2_ and act as an important climate driver. Over the last two decades there has been increasing interest from scientists, governments and land managers in the role that soils play in controlling atmospheric CO_2_^1^. IPCC guidelines exist specifically to calculate the carbon stocks and sequestration potential of soils under different biomes and management regimes^3^ and are regularly used along with other models of soil carbon dynamics to determine national carbon stocks and sequestration potential^6, 7^.

As the most actively managed, agricultural soils are the most amenable to sequestration of atmospheric CO_2_. IPCC has suggested that globally, agricultural soils could sequester 1400–2900 Tg of CO_2_ equivalents annually for 50–100 years^8^, while Lal^1^ suggested ‘normal’ rates of SOC sequestration are in the order of 300–500 kg C ha^−1^ yr^−1^ either through reductions in or elimination of tillage, or application of organic wastes^2, 9–13^. Alternatively, the abandonment of cropping and establishment of grassland or forest also has the potential to sequester significant atmospheric CO_2_^13–15^. Much of the literature in this field supports these views and the assumptions in IPPC guidelines continue to be used to calculate and promote national carbon sequestration potential of agricultural soils^6, 16, 17^. As such, IPCC guidelines have the potential to have a significant impact on climate policy. However, there is huge uncertainty associated with some of the assumptions in the IPCC guidelines and IPCC recommends use of country specific data in preference to IPPC default guidelines when calculating carbon stocks and sequestration potential^3^. Attempts to model carbon sequestration potential in specific regions with specific farming types have nevertheless also suffered similar wide levels of uncertainty^18, 19^. Indeed the whole basis of the paradigm that agricultural soils can sequester significant atmospheric carbon has been questioned^11, 12, 20^ and there are clear methodological problems with some of the supporting science^20–22^. If policy is to be effective in capturing the potential for agricultural soils to sequester atmospheric CO_2_ then actual carbon sequestration potential and the key controlling factors need to be established.

Whist the literature contains extensive analysis of changes in SOC associated with changes in soil tillage^11, 23–25^ similar data associated with conversion of cropland to grassland is less extensive^13^. Data which does exist suggests large variation in carbon sequestration potential on small scales, associated with soil moisture gradients, soil physio-chemical characteristics and plant species^26, 27^ or is based on modelling of large scale datasets and comes with large uncertainties^13^. This being the case, it is important to understand the carbon sequestration potential of converting cropland to grassland and the key drivers at the scale of land management rather than the broad scale calculations used in IPCC guidelines if it is to be used as a policy instrument for mitigating climate change.

Although widespread conversion of cropland to grassland for carbon sequestration purposes has not yet happened, there are a number of examples globally of schemes to convert land from cropping to grassland, usually in response to land degradation. These include the ‘Grain-for-Green’ program initiated in China, in 2000^28^ and a number of prairie restoration programs in the USA, notably the Conservation Reserve Program (CRP), initiated in 1985^29^. In Europe, land abandonment has occurred on a significant scale in parts of the former Soviet Bloc^30, 31^ and in Western Europe an arable land ‘set-aside’ scheme was initiated in 1988 in response to overproduction of commodity crops in EU countries. This required arable land to be removed from production, either for one year (rotational set-aside) or for a longer period (non-rotational set-aside) as part of the EU farm subsidy program. Initially it was a voluntary scheme, but after 1992 it became compulsory. The area of land set-aside varied from year to year during the scheme, with an average level of 10% across the EU and 8% in England, with around 60% of the English area in non-rotational set-aside^32^. Although compulsory set-aside ended in 2008, many areas of non-rotational set-aside were retained under other agri-environment schemes^33^. Although none of these land abandonment programs were initiated with the aim of carbon sequestration, there has been a great deal of retrospective interest in their carbon sequestration potential as part of national climate change mitigation actions^6, 7, 15, 28, 34^. However, estimates of their carbon sequestration potential vary enormously. King *et al*.^35^ estimated that set-aside field margins on arable land in England could sequester 490–734 kg C ha^−1^ yr^−1^, while Freibauer *et al*.^36^ suggested that sequestration potential of set-aside across the EU15 was less than 400 kg C ha^−1^ yr^−1^. Soils under the Grain-for-Green program in China have been estimated to sequester 430 kg C ha^−1^ yr^−1^ when converted to grassland^15^. Sperrow^6^ attempted to improve estimates of farmland potential to sequester CO_2_ using IPCC guidelines, estimating annual sequestration potential of land in the CRP program of 270–360 kg C ha^−1^ yr^−1^, depending on tillage practice prior to enrolment. Landscape scale estimates suffer from similarly large ranges, with soils converted to grassland under the US CRP program having been estimated to sequester between 4.4 Tg C yr^−1^ and 11 Tg C yr^−1^, but with an outlier estimate of 29 Tg C yr^−1^ ^6^.

While the carbon sequestration potential of agricultural land remains uncertain, it is now widely accepted that soil microorganisms play an important role in in mediating carbon sequestration in soils and by inference can have a significant influence on climate change^37^. Therefore, understanding the process involved in carbon sequestration requires an understanding of microbial community structure and function. However, we have very limited understanding of the response of the microbial community (biomass, structure and diversity) to changes in land-use, particularly the timescales over which they occur. Nevertheless, we can be reasonably certain that the response of microbial communities and thus the carbon sequestration potential of soils is likely to depend on the ecosystem in question, will be moderated by changes in quantity/quality of carbon inputs^37, 38^ and is thus operative on a completely different scale to calculations made using IPPC guidelines.

The aim of this work was therefore to determine the scale of changes in SOC stock associated with land use change from arable cropping to minimally managed grassland at the field scale, and establish any soil physico-chemical and microbial community changes associated with this land use change, which could be key drivers of changes in SOC, using non-rotational set-aside in England as the model system. Fourteen sites were identified where grassland had been established under the set-aside scheme between 6 and 17 years previously, which could be compared with land under continuous arable production, either within the same field or an adjacent field with the same soil type. Soil carbon stocks, physico-chemical characteristics and microbial community structure were determined. In addition, two replicated experiments were established at a single site and used to establish changes in SOC distribution through the profile, soil physico-chemical characteristics and microbial community structure during transition between grassland and arable cropping in order to establish direction of change in soil processes.

## Results

### Soil physico-chemical parameters

#### Replicated land use change experiments at Wellesbourne, UK

*Set-aside to arable conversion:* There was a significant change in the distribution of SOC upon change of management from set-aside to cropping, with sampling depth (P < 0.001), and the interaction between depth and cropping (P < 0.001) being significant (Table 1). In set-aside plots SOC concentration was significantly higher in the top 10 cm than at 10–30 cm, ploughing up the set-aside and returning it to cropping destroyed this stratification with carbon concentration the same throughout the top 30 cm in the arable plots. Mean SOC through the top 30 cm was not affected by cropping (P = 0.921).

**Table 1:** Soil parameters in set-aside to arable replicated land use change experiment.

	SOC (%)		N (%)		(µg g^−1^)						pH	
					NO_3_^−^ N		*Total P*		*Olsen P*			
Depth	0–10 cm	10–30 cm	0–10 cm	10–30 cm	0–10 cm	10–30 cm	0–10 cm	10–30 cm	0–10 cm	10–30 cm	0–10 cm	10–30 cm
Treatment												
SAA	2.06a	2.03a	0.170a	0.165a	10.6a	12.0a	605a	560a	48a	43a	5.8a	5.9a
	*0.40*	*0.28*	*0.043*	*0.030*	*3.2*	*1.9*	*86*	*73*	*7*	*8*	*0.5*	*0.7*
SSA	1.97a	2.05a	0.162a	0.163a	9.0a	6.0a	783a	641a	59a	53a	5.8a	5.9a
	*0.21*	*0.28*	*0.019*	*0.023*	*3.3*	*1.7*	*344*	*159*	*13*	*18*	*0.9*	*0.9*
SSS	2.60a	1.58b	0.202a	0.128b	4.1a	3.4a	674a	577a	67a	40b	5.7a	5.9a
	*0.54*	*0.32*	*0.048*	*0.026*	*6.6*	*1.0*	*47*	*98*	*14*	*13*	*0.7*	*0.8*
Significance												
*Depth*	<0.001		<0.001		0.477		0.027		<0.001		<0.001	
*Treatment*	0.921		0.401		0.008		0.137		0.373		0.898	
*Interaction*	<0.001		<0.001		0.335		0.688		<0.001		0.195	

Means with SD in italics, letters indicate signify differences between depths within each treatment. Cropping - SAA 12 years set-aside followed by two years arable (n-5), SSA 13 years set-aside one year arable (n-5), SSS continuous set aside for 14 years (n-5).

A similar response was observed with total N. In contrast, NO_3_^–^N showed a significant treatment effect, with increasing concentration the longer it had been in cropping. NO_3_^–^N was significantly higher in the plots after two years of arable cropping (11.3 µg g^−1^) than under continuous set aside (3.7 µg g^−1^) while plots under one year of arable cropping showed a trend for more NO_3_^–^N, (7.5 µg g^−1^), there was no depth effect. Extractable P showed a similar trend to SOC and total N. pH was not influenced by treatment, but there was a significant influence of depth, pH at 0–10 cm (5.75) being lower than at 10–30 cm (5.92).

*Arable to set-aside conversion*: In the arable to set-aside conversion (Table 2) there was no significant effect of cropping on SOC concentration (P = 0.685), but the effect of sampling depth was significant (P < 0.001), with slightly higher SOC concentration in the surface 0–10 cm, though the interaction was not significant (P = 0.179). Cropping had no significant effect on total soil N (P = 0.499), but did have a significant effect on soil NO_3_^–^N (P < 0.001) with a significant reduction in NO_3_^–^N on conversion from arable to set-aside, with a particularly large reduction in year one, NO_3_^–^N falling from 5.06 µg g^−1^ to 1.14 µg g^−1^ in the first year. The effect of sampling depth on NO_3_^–^N was not significant (P = 0.554). Neither total nor extractable P or soil pH were significantly affected by cropping and there were no significant depth effects.

**Table 2:** Soil parameters in arable to set-aside replicated land use change experiment.

	SOC (%)		N (%)		(µg g^−1^)						pH	
					NO_3_^−^ N		*Total P*		*Olsen P*			
Depth	0–10 cm	10–30 cm	0–10 cm	10–30 cm	0–10 cm	10–30 cm	0–10 cm	10–30 cm	0–10 cm	10–30 cm	0–10 cm	10–30 cm
Treatment												
AAA	1.58a	1.51a	0.10a	0.10a	5.0a	5.1a	1269a	1391a	93a	94a	7.5a	7.5a
	*0.09*	*0.09*	*0.005*	*0.015*	*2.8*	*1.1*	*71*	*291*	*13*	*16*	*0.3*	*0.2*
AAS	1.66a	1.51a	0.10a	0.09a	1.5b	0.8b	1276a	1223a	97a	99a	7.5a	7.5a
	*0.23*	*0.14*	*0.007*	*0.004*	*0.8*	*0.5*	*81*	*63*	*12*	*14*	*0.3*	*0.3*
ASS	1.66a	1.50a	0.10a	0.10a	1.0b	0.2b	1215a	1292a	101a	101a	7.4a	7.4a
	*0.15*	*0.15*	*0.003*	*0.005*	*0.7*	*0.2*	*79*	*143*	*9*	*7*	*0.2*	*0.2*
Significance												
*Depth*	<0.001		0.308		0.544		0.214		0.988		0.276	
*Treatment*	0.85		0.499		<0.001		0.300		0.471		0.506	
*Interaction*	0.179		0.354		0.733		0.410		0.752		0.836	

Means with SD in italics, letters indicate signify differences between depths within each treatment. Cropping AAA - continuous arable (n-5), AAS - one year set-aside after > 30 years arable (n-5), ASS – two years set-aside after >30 years arable (n-5).

#### Landscape scale experiment

**Figure 1:**
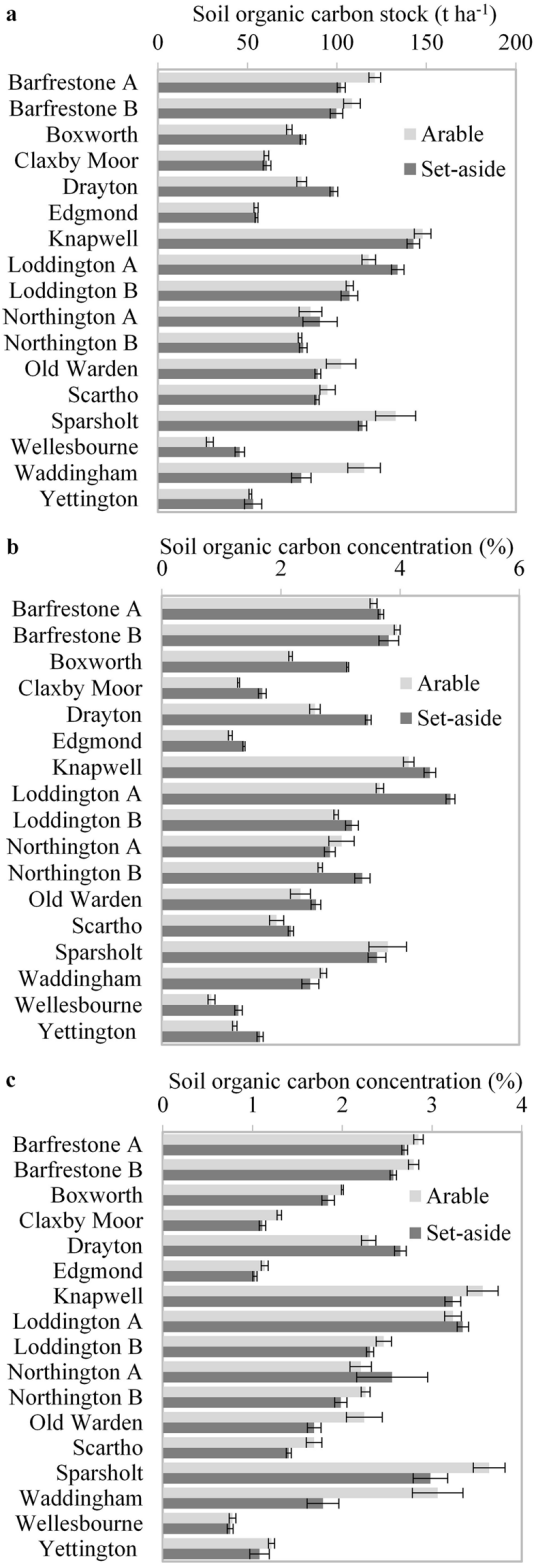
Soil organic carbon under arable and set aside management. Fourteen sites (17 paired comparisons) in England. (**a**) Carbon stock from 0–30 cm. (**b**) Soil organic carbon concentration from 0–10 cm. (**c**) Soil organic carbon concentration from 10–30 cm. Columns represent means of five plots. Error bars represent +/− one standard error.

There were large differences in SOC stocks between sites, with the highest (152 t ha^−1^) more than three times the lowest (39 t ha^−1^) (Fig. 1a). There was however, no significant difference between carbon stocks under cropped and set-aside areas (P = 0.64) and notably there was there was no effect of age of set-aside (Table S2), suggesting that the difference between set-aside and arable was not increasing with age. When individual sites were analysed separately using the five SOC analyses taken at each site (i.e. pseudo replicates as true replicates), then three sites did have significantly higher SOC stock in the set-aside (Boxworth, Drayton, Loddington A) but two sites had significantly more in the arable (Barfrestone A, Waddingham).

Dividing soils into 0–10 and 10–30 cm depths and considering SOC concentration revealed significant differences in distribution of SOC, similar to those seen in the replicated experiment (Table S3). Sampling depth had a significant effect on SOC concentration (P < 0.001) and there was a significant interaction with cropping (P < 0.001), though as with consideration of carbon stocks, there was no significant difference between arable and set-aside (P = 0.393). Soils under set-aside had a higher concentration of SOC at 0–10 cm (2.87%) compared with soils under arable cropping (2.53%), with 13 of 17 paired sites having a higher concentration in the set-aside soils (Fig. 1b). However at 10–30 cm, soils under set-aside had a lower concentration of SOC, (1.99% compared with 2.21%), with 14 out of 17 paired sites having a higher concentration in the arable soils, (Fig. 1c) indicating a redistribution of SOC in set-aside, rather than an overall increase.

Other soil characteristics compared on a concentration basis showed a similar pattern as the replicated experiments (Table 3). Total N was not significantly different between arable and set-aside (P = 0.775) although there was a significant depth effect (P < 0.001) and the interaction between depth and cropping was significant (P < 0.003), with set-aside having a more stratified distribution of total N. NO_3_^–^N was significantly different between set-aside and arable, with a significant depth effect (both P < 0.001). NO_3_^–^N concentration was significantly higher in arable soils at both sampling depths. Unlike for the replicated experiments, total P was significantly influenced by cropping, with more P in arable than set-aside and there was also a significant depth effect, with more total P at 0–10 cm, though the interaction was not significant. Extractable P also differed significantly between set-aside and arable (P = 0.037), with more extractable P in arable soils and more in soil from 0–10 cm, though the interaction was not significant (P = 0.751). pH did not differ between cropping types (P = 0.594).

**Table 3:** Soil parameters in set-aside and arable cropped land at 14 sites (17 paired comparisons) in England.

	%C		N%		(µg g^−1^)						pH	
					NO_3_^−^		Total P		Olsen P			
Depth	0–10 cm	10–30 cm	0–10 cm	10–30 cm	0–10 cm	10–30 cm	0–10 cm	10–30 cm	0–10 cm	10–30 cm	0–10 cm	10–30 cm
Cropping												
Arable	2.58	2.28	0.30	0.26	48.8	16.4	812	695	44.6	33.7	6.9	7.2
	*1.04*	*0.86*	0.15	0.12	*46.1*	*8.2*	*343*	*271*	*30.4*	*26.4*	*0.79*	*0.68*
Set-aside	2.92	2.06	0.32	0.24	15.2	10.0	724	610	39.4	26.7	6.9	7.1
	*1.05*	*0.81*	0.14	0.11	*9.4*	*6.9*	*280*	*247*	*24.7*	*23.5*	*0.90*	*0.80*
Significance												
*Crop*	0.383		0.775		<0.001		0.002		0.037		0.594	
*Depth*	<0.001		<0.001		<0.001		<0.001		<0.001		0.001	
*Interaction*	<0.001		0.003		0.001		0.953		0.751		0.813	

Values represent means of five plots with SD in italics.

### Microbial community analysis

#### Replicated land use change experiments at Wellesbourne, Warwickshire, UK

*Set-aside to arable conversion:* Fifty Phospholipid Fatty Acids (PLFAs) were quantified in each of the soil samples. Combined data from all the PLFAs was used to determine total microbial biomass^39^. Total PLFA in the 0–10 cm depth samples was 63.6, 28.7 and 38.7 nmol g^−1^ soil in set-aside, and one and two years following conversion from set-aside to arable respectively. In the 10–30 cm depth samples, total PLFA were 37.9, 33.3 and 34.3 nmol g^−1^ soil in set-aside and one and two years following conversion from set-aside to arable respectively. The difference in total PLFA between set-aside and arable was significant in the 0–10 cm depth samples (P = 0.009), but not in the 10–30 cm depth samples (P = 0.891).

**Figure 2:**
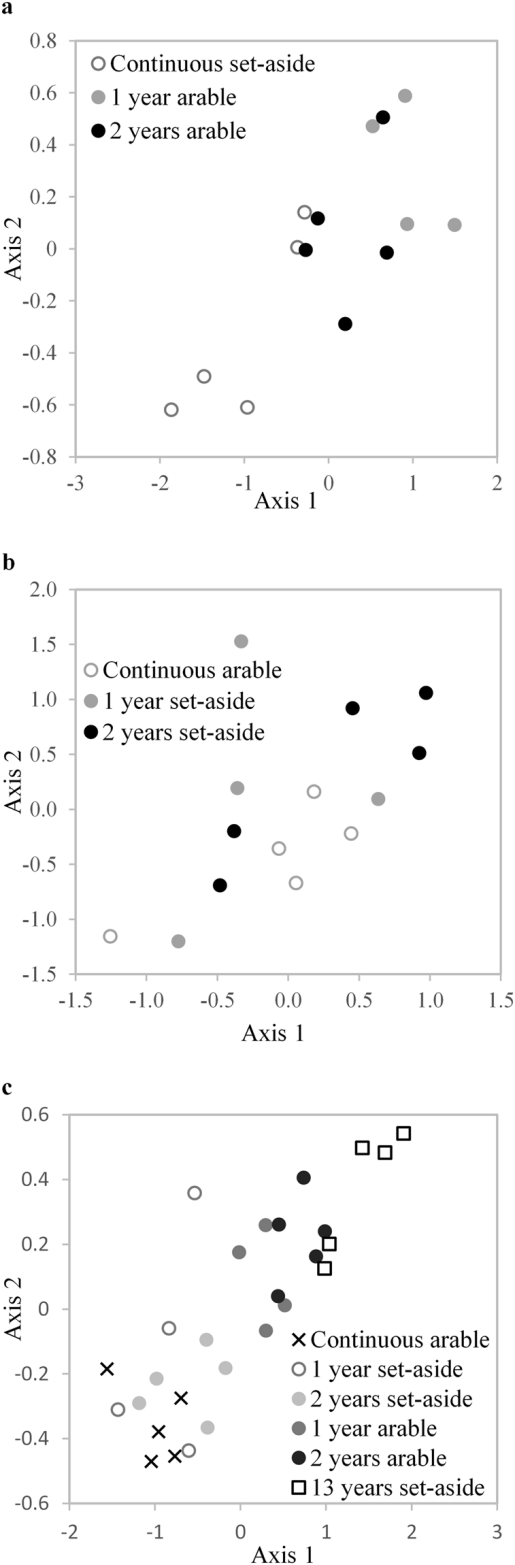
NMDS plot of PLFA profiles. Soils sampled from 0–10 cm from individual trial plots (n-5). (**a**) Under continuous set-aside for 14 years or after reversion to arable cropping for one or two years. Final stress for 2-dimensional solution 1.02, final instability <0.001, number of iterations 207. Variation in distance matrix represented by: axis 1, 93.9%; axis 2, 3.6%. (**b**) Under continuous arable for more than 30 years or after conversion to set-aside for one or two years. Final stress for 2-dimensional solution 3.79, final instability <0.001, number of iterations 123. Variation in distance matrix represented by: axis 1, 45.9%; axis 2, 52.0%. (**c**) All plots at different stages of conversion from arable to set-aside or set-aside to arable analysed together. Final stress for 2-dimensional solution 1.68, final instability <0.001, number of iterations 76. Variation in distance matrix represented by: axis 1, 97.2%; axis 2, 1.3%.

The PLFA fingerprint of 0–10 cm set-aside soil and 0–10 cm soil which had reverted to arable cropping is shown in Fig. 2a. MRPP analysis showed that one or two year arable populations were significantly different to continuous set-aside (P = 0.004 and 0.013 respectively) with clear separation along axis 1. SIMPER analysis was used to identify the PLFAs contributing to the separation between treatments. PLFAs 18:1w7, 16:0 and 18:1w9 were found to contribute 15.2, 10.7 and 8.6% to the dissimilarity between arable and set-aside plots after two years in arable cropping. All showed a large reduction compared with the overall mean reduction in PLFAs. PLFA 18:1w9 has been identified with fungi^40^ and 18:1w7 and 16:0 with bacteria including methanotrophs (18:1w7)^41^. Other PLFAs which contributed over 5% to the dissimilarity were 16:1w7c, 16:1w5, 19:0cy, phthalate and C15:0i. 16:1w5 has been associated with arbuscular mycorrhizal (AM) fungi^42, 43^ and declined by more than 50% on reversion to arable. PLFA profiles in soils from 10–30 cm were not separated by NMDS and were not significantly different.

*Arable to set-aside conversion*: Total amounts of PLFA in this field were considerably lower than in the field undergoing reversion from set-aside to arable. The total amount of PLFA in soil remaining under arable and soil which had been converted to set-aside for one and two years was 12.0, 14.5 and 16.2 nmol g^−1^ respectively at 0–10 cm depth and 15.2, 11.9 amd 13.9 nmol g^−1^ respectively at 10–30 cm. Despite the increasing trend at 0–10 cm there was no significant difference in total PLFA. NMDS analysis is shown in Fig. 2b. There was no significant difference in the PLFA fingerprint between the continuous arable and set-aside. MRPP similarly indicated no significant change to the PLFA profile after one or two year’s reversion at 10–30 cm depth.

When 0–10 cm depth soil PLFA profiles of all plots in arable to set-aside and set-aside to arable were compared using NMDS (Fig. 2c) there was a clear progression from 14 years set-aside to continuous arable along axis 1. MRPP analysis showed that all the treatments in the set-aside to arable experiment were significantly different (P < 0.01) to all the treatments in the arable to set-aside experiment, indicating that microbial communities in soils where management had changed were more similar to communities under the original management than communities representative of the new management.

#### Landscape scale experiment

Across the 17 pairs of set-aside and arable, total PLFA abundance was significantly higher in set-aside soil relative to arable, 52.9 and 31.4 nmol g^−1^ soil respectively (P < 0.001). Total abundance of PLFAs was higher in the set-aside at all sites, although differences were small at some sites, notably Northington A, but very large at others, with more than double the total PLFAs in set-aside as arable at Barfrestone A, Boxworth, Edgmond, Loddington A, Northington B, Old Warden and Wellesbourne.

Across the 17 pairs, only four of the 50 PLFAs were more abundant in arable soils and none have been associated with specific microbial groups. Of the other PLFAs, 13 showed no significant difference between set-aside and arable, with the other 33 being significantly more abundant in set-aside. A more informative analysis could be gained by examining those PLFAs that were higher or lower as a proportion the total PLFAs. There were eight with greater than 1.25 as much of the PLFA in set-aside as arable. Five of these have not been associated with any specific microbial group, but three; 16:1w5, 18:2(9,12) and 20:5w3 have all been associated with fungi^29, 44^ (16:1w5 with AM fungi^42^). All were significant more abundant in set-aside. Several PLFAs were present at less than 0.75 as much in set-aside as arable, but none of these differences were statistically significant and none have been associated with any specific microbial group. All three PLFAs associated with actinomycetes 10Me16:0, 10Me17:0, and 10Me18:0^40^ were a larger proportion of total PLFAs in arable than set-aside soils. 10Me17:0, and 10Me18:0 were significantly more abundant in the arable soils. 10Me16:0 was not significantly different, but only made up 7% of the actinomycete PLFA markers in arable and 13% in set-aside.

**Figure 3:**
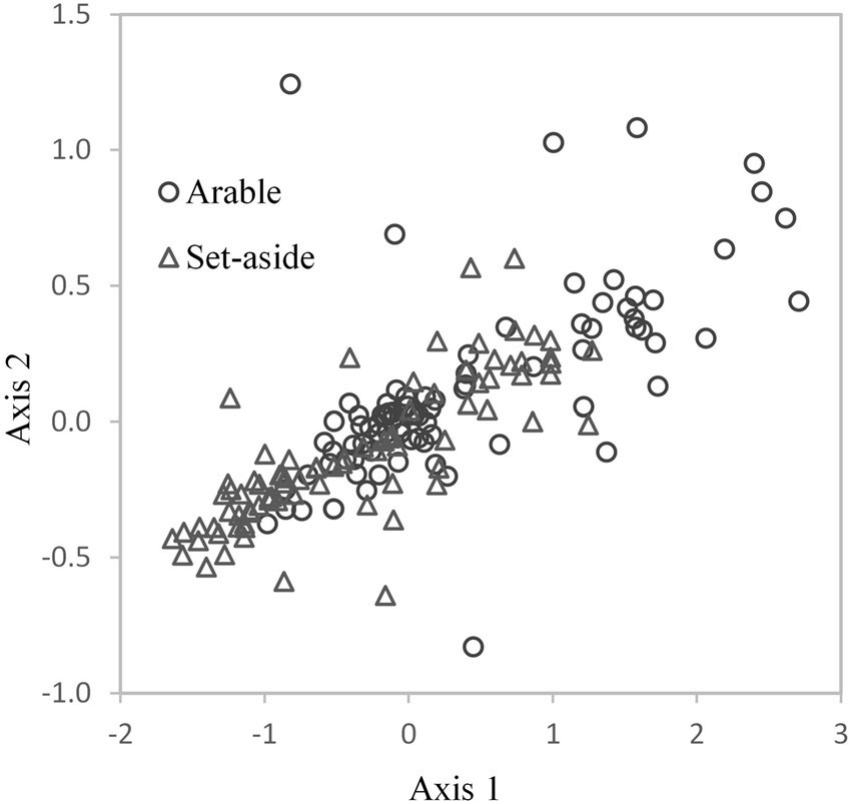
NMDS plots of PLFA profiles. Soils sampled 0–10 cm in set-aside and arable cropped areas of fields or whole fields at 14 sites (17 paired comparisons) in England (n−168). Final stress 4.95, final instability <0.001, number of iterations 243, Variation in distance matrix represented by: axis 1, 94.0%; axis 2, 3.2%.

NMDS plot of all paired sites demonstrated that there was no distinct PLFA profile in arable or set-aside soil, with overlap between arable and set-aside sites (Fig. 3). However, despite the overlap there was separation of arable and set-aside sites along axis 1, and MRPP confirmed that there was a significant difference (P < 0.001). When NMDS plots were prepared for individual pairs, in most cases set-aside and arable were clearly separated (See Fig. S1), with MRPP demonstrating significant differences at 14 of the 17 pairs.

SIMPER analysis was used to identify the key PLFAs responsible for separating set-aside and arable within the NMDS plots. Broadly the same PLFAs were responsible for separating set-aside and arable when all farm sites were analysed together, and when individual farm sites were examined. In the case of all farm sites together, the key PLFAs (Table S4) were 18:1w7, 16:0, 16:1w7c and 19:0cy, which have been associated with Gram + ve and Gram –ve bacteria and 18:1w9, which has been associated with fungi^40^. At individual farm sites 18:1w7 was again the dominant PLFA in terms of contribution to dissimilarity. However, at ten sites 16:1w5, associated with AM fungi^42^, contributed more than 5% dissimilarity. It contributed 4.92% to dissimilarity in the SIMPER analysis in the over-site analysis.

## Discussion

Although carbon sequestration in agricultural soils has been promoted as a climate change mitigation measure, the true capacity for this is under dispute^1, 20, 45, 46^. Our results suggest abandoning arable land in N.W. Europe and allowing it to revert to grassland would result in very little carbon sequestration in the short to medium term. This result is at odds with other work showing very high rates of SOC accumulation in similar circumstances^46–48^. Some instances where large increases in SOC stocks have been reported can be explained by a failure to measure to sufficient depth. Mensah *et al*.^49^ only measured to 15 cm, McLauchlan *et al*.^50^ Hamer *et al*.^51^ and Hirsch *et al*.^52^ to 10 cm and McKinley *et al*.^29^ to 5 cm. Our work demonstrates that this is not sufficient. We recorded a significantly different distribution of SOC between set-aside and arable, with SOC concentrated in the top 10 cm in the set-aside soils in both the replicated and landscape scale experiments. Had we restricted our analyses to 10 cm we would also have found a significant increase in SOC stock in the landscape scale experiment. As here, Kucharik *et al*.^53^ examined 39 paired arable and restored CRP grassland sites in Wisconsin and found that while there was a significant increase in SOC stock in the top 5 cm, through the whole top 25 cm there was no net increase. Failure to measure to sufficient depth was a key criticism of IPCC guidelines by Powlson *et al*.^20^. Carbon sequestration in deeper soil layers, below cultivation depth, may occur in response to land use change, but the evidence is contradictory and the capacity uncertain^45^. Any sequestration below cultivation depth in the set-aside is unlikely to have been significant.

As well as methodological issues, there may be a real limitation in the potential for abandoned arable land in N.W. Europe to sequester CO_2_. The key changes associated with abandonment are an end to cultivation and a change in residue inputs. Cessation of cultivation has a marginal impact on SOC stock, particularly where reduced tillage is already practiced, as is common in NW Europe^11, 12^. Increased carbon input through perennial vegetation cover would therefore be the key driver in any increase in SOC. However, Western European agriculture is high input with high crop yields (UK wheat yield > 7.5 t ha^−1^ ^54^,). Above ground crop residue yields are approximately 60% of grain yield, meaning large inputs of residue carbon into soils. Yields of unimproved grassland in the UK range from only 2 to 8 t dry matter ha^–1^ ^55^, and so after cropping ceases, residue inputs may actually fall, limiting carbon sequestration potential. In contrast, on marginal land^50^, in semi-arid regions^56^ or in areas with low input agricultural^31^ crop productivity and residue inputs are low (North American wheat yields < 3 t ha^−1^ ^54^,) and therefore more similar to residue inputs after land is abandoned. Furthermore, in the establishment phase of unmanaged grassland, residue input is likely to be further restricted. Kalinina *et al*.^31^ observed incomplete cover associated with ruderal plant species during natural reversion of abandoned cropland in Russia, with a resultant low accumulation rate of SOC, over eight years. Matamala *et al*.^57^ reported that vegetation C took 13 years to recover to natural levels in a restored prairie sequence, while Boatman *et al*.^58^ recorded high levels of bare ground in the first two years of set-aside in 287 fields in the UK. Farm records indicated that initial establishment of vegetation was often slow at farm sites in the landscape study, and despite the fact that the grass was sown in the experiments at Wellesbourne, after two years ground cover was incomplete, supporting these other studies. Given the fact that age of set-aside was not a significant factor in our landscape study it seems likely that a much longer period of land abandonment is required under these conditions to achieve measurable net carbon sequestration.

Slow establishment and low productivity is likely to be driven by low soil mineral N and significant competition from soil micro-organisms for that N^59^. We recorded significantly lower concentrations of NO_3_^–^N in set-aside soils relative to arable in the landscape study, while in the replicated land use change experiments NO_3_^–^N declined by almost 80% in the first year after cropping ceased, as a consequence of a halt to applications of N fertiliser. Zhang *et al*.^15^ reported low vegetation productivity in abandoned cropland, associated with low soil N, with low levels of mineral N in re-vegetated soils also being reported by Reeder *et al*.^60^. This will put an immediate limit on grass productivity. Modelling by Soussana *et al*.^46^ similarly suggested N limitation would reduce the productivity of grassland on abandoned arable land, limiting carbon sequestration potential. Recovery in soil N is likely to be slow. Kalinina *et al*.^31^ reported soil N concentrations in abandoned arable land only recovered after 37 years, with Matamala *et al*.^57^ reporting vegetation levels of N in restored prairie recovering to natural levels after 21 years. Given the apparent role of low soil N in limiting sequestration, application of N fertiliser when grass is established could boost sequestration. Reeder *et al*.^60^ compared re-vegetation of cropland in Wyoming with and without supplementary N. On clay soil, a significant increase in SOC was achieved in the first five years only with supplementary N fertiliser. A more sustainable boost to soil N and thus carbon sequestration may be achieved through using more diverse species^26, 61^. Set-aside vegetation at the farm sites used here was a mixture of natural re-vegetation and sown swards, usually with a single grass species, potentially limiting sequestration. If productivity was brought up to levels achieved in managed grassland, modelling suggests net carbon sequestration could be achieved in N.W. European conditions^19^.

While we did not record a significant increase in SOC stocks, we did record a significant difference in soil microbial populations between set-aside and arable in the landscape study. This difference was evident in the first year of management change in the replicated experiments, although evidence from Fig. 2c suggest microbial populations retained characteristics of the previous management even after two years, similar to the observation of Hirsch *et al*.^52^. While caution must be exercised when attributing individual PLFA to specific microbial groups^43^, it appeared that there was a lower relative abundance of actinomycetes and more fungi in set-aside. Both general fungal PLFAs, 18:2(9 12) and 20:5w3^29, 44^ and the AM fungal associated PLFA 16:1w5^42^, were higher relative to total PLFA in the set-aside soils. Tillage has a large detrimental effect on AM fungi^62^ and so a cessation of tillage under set-aside would be expected to have this impact. Grassland also often has a higher ratio of fungi to bacteria^63, 64^ particularly where the grassland is unfertilised^65^ although McKinley *et al*.^29^ recorded a relative decline in fungal PLFAs with age of restored prairie and Strickland & Rousk^66^ caution against broad generalisations about bacterial to fungal ratios in different management systems. Nevertheless, the decline in bacterial dominance seen here would be expected to enhance carbon sequestration as fungi have higher carbon assimilation efficiencies and fungal cell wall carbon polymers are much more resistant to decomposition than those in bacterial cell membranes. As a result, in ecosystems dominated by fungi, soil respiration rates are typically low, which increases the potential for carbon sequestration^67^. Furthermore, SOC is richer in aromatic compounds under a grassland than under cereals, which confers on it a greater ability to resist degradation^68^. Therefore the change we observed in the microbial community under set-aside should enable enhanced SOC sequestration, even if the change in community structure was still incomplete after two years.

Given our results, as a policy measure aimed at sequestration of atmospheric CO_2_ and mitigation of climate change, untargeted reversion of arable land to grassland does not seem to be an efficient option under U.K. conditions. As crop rotations, agronomic practices and climate are similar across N.W. Europe, this result is likely to hold across much of this region and potentially to other parts of the world with similar climate and agricultural practices. Although a more targeted approach to land abandonment, particularly focusing on poorly drained sites^27^ and utilising sward species mixtures with high productivity potential under nutrient limitation, may improve carbon sequestration potential, there is another critical factor to consider in policy terms. That is the long-term management of land abandonment for carbon sequestration. If as this work indicates, short to medium term carbon sequestration is not likely to be significant in many cases, policy must aim at long-term land abandonment. This is even more critical given that even short term cultivation of abandoned land is likely to result in the rapid loss of any sequestered carbon^46, 47, 60, 69^. Such long-term abandonment of highly valuable farmland in Western Europe is likely to be difficult and potentially costly to achieve, with farmers generally reluctant to commit to long-term management changes aimed at carbon sequestration^17^. This is in addition to the implications of land abandonment for food production and the viability of farm businesses. As such, widespread land abandonment aimed at carbon sequestration can only be undertaken after careful consideration of the merits of such a policy compared with other climate change mitigation options, combined with appropriate management on the ground.

## Methods

One of the limitations of many studies examining SOC sequestration in cropland and the associated microbial community dynamics is their failure to address appropriate spatial and temporal scales at which management of these soils occurs. Either studies are very limited in scope and address a single site, which may not be representative or use a meta-analytical approach, with large numbers of sites over multiple environments which have employed differing methodologies and where the impact of environmental factors may be lost. We sought to overcome these limitations by combining short-term replicated land use change experiments at a single site with a landscape scale study of multiple paired arable and set-aside sites, encompassing a range of soil conditions, timescales, crop agronomy and grass sward, representing a single climatic zone in England with similar farm management.

### Experimental sites

#### Replicated land use change experiments

Two replicated field experiments were set up in September 2008 at Wellesbourne, England (Lat N52:12:00 Long W1:36:34), to convert land from set-aside to arable cropping and vice versa (Table S1). Experiments were set up in Water Meadows field, which had been in non-rotational set-aside for 12 years, with grass managed by annual mowing, and Long Close field, which had been in cereal rotation for at least 30 years. In both fields, 15, 12 m × 3 m plots were arranged in three blocks of five. In Water Meadow field, conversion from set-aside to arable was conducted in five randomly selected plots in October 2008 and a further five in 2009 by ploughing the grass sward and harrowing prior to sowing wheat. Thus giving five plots converted for one year and 5 plots converted for two years. Winter wheat was grown on arable plots and was managed according to conventional local farm practice, with applications of N, P and K fertiliser, herbicide, molluscide and insecticide. The other plots remained as set-aside. In Long Close field, following harvest of the previous wheat crop, the field was ploughed and five randomly selected plots were converted to set-aside by seeding with rye grass (*Lolium* spp.) in October 2008 and a further five in 2009. Thus giving five plots converted for one year and 5 plots converted for two years. The plots were managed as described above for set-aside. Plots remaining in arable cropping were planted with winter wheat and managed as above.

After two years soils were sampled for SOC and microbial populations from each of the 15 plots in Water Meadow and Long Close fields, taking 20 soil cores from each plot to a depth of 30 cm using a 5 cm diameter auger. Cores were divided into 0–10 and 10–30 cm depth in the field and pooled for each sampling depth. Sampling took place in the September after the harvest of wheat.

#### Landscape scale measurements

To account for differences in management and environment, a multi-site landscape scale experiment was conducted to investigate relative differences in SOC and soil microbial populations between set-aside and continuously cropped soil, using the paired field approach. Samples were taken from two immediately adjacent fields or areas within a field where part of the field was managed under set-aside and the remaining area continued to be cropped.

A total of 14 locations were identified across England (Table 4), with three sites allowing comparison of two paired comparisons, to give 17 paired comparisons in total. The form of the set-aside within cropped fields varied, but included blocks up to one ha in area and field strips between 6 and 20 m wide, both along margins and within the main field area. Age of the set aside varied between 6 and 17 years. The sites were geographically widespread, and included soils of different textures (soils hand textured in field), from clay to sandy loam, including some shallow soils (<40 cm) overlaying chalk or limestone. In some cases, set-aside had been established by sowing a grass seed mixture while in other cases vegetation was allowed to regenerate naturally. In all cases set-aside was mowed annually. All cropped areas were in a conventional arable rotation with a mixture of small grains and oilseed rape (canola) with occasional crops of field beans (*Vicia faba*), with management employing standard N,P and K fertiliser additions and crop protection chemicals. The crops grown, management of the sites, both cropped and set-aside areas, and the soil types are typical of N.W. Europe and the results are therefore more widely applicable than England.

**Table 4:** Sampling locations used in landscape scale assessments with soil texture, assessed in the field by hand texturing and years set-aside had been established.

Site	Approximate location		Soil type	In field or between field comparison	Set aside age
	Latitude	Longitude			
Barfrestone A + B	N 51°12′	E 1°14′	Silt	In/In	14
Boxworth	N 52°14′	W 0°02′	Clay	Between	16
Claxby Moor	N 53°26′	W 0°22′	Clay loam	In	10
Drayton	N 52°05′	W 1°45′	Clay	Between	6
Edgmond	N 52°46′	W 2°26′	Silt	In	6
Knapwell	N 52°15′	W 0°02′	Clay loam	In	8
Loddington A + B	N 52°36′	W 0°49′	Clay loam	In/In	17
Northington A + B	N 51°08′	W 1°11′	Silt/Clay	In/In	8
Old Warden	N 52°05′	W 0°20′	S. loam	Between	10
Scartho	N 53°32′	W 0°04′	Loam	Between	11
Sparsholt	N 51°05′	W 1°23′	Loam	In	12
Waddingham	N 52°27′	W 0°32′	Silt	Between	7
Wellesbourne	N 52°12′	W 1°36′	S. loam	In	7
Yettington	N 50°39′	W 3°20′	S. loam	In	15

Soil sampling took place in March and April 2008. At each location 5 contiguous 10 × 10 m areas within the set-aside and cropped areas were sampled (except in the case of field strips, in which 4 × 25 m areas were sampled). When the comparison was made between different fields the sampling area was located centrally within each field. When the comparison was made within fields the set aside and cropped areas were adjacent, separated by at least 10 m to avoid field headlands. From each plot 20 soil cores were collected from the top 30 cm using a 5 cm diameter soil auger and divided into 0–10 and 10–30 cm depth in the field and pooled. Bulk density was measured separately at 0–10 and 10–30 cm depth in each plot using 10 cm diameter steel lidded tins, giving five replicates per depth within each treatment at each site.

### Soil physico-chemical analysis

The soils were stored at 4 °C before being sieved (<3 mm) and then analysed for total C and N content (Leco C/N analyser) pH, NO_3_-N and total and extractable (Olsen) P. Soil carbon stocks were calculated on an equivalent soil mass basis in order to account for differences in soil bulk density between arable and set aside treatments^21^.

### Microbial community structure

Phospholipid fatty acid (PLFA) analysis was used to determine microbial abundance and community structure using the extraction method of Bligh and Dyer^70^ as modified by Frostegaard *et al*.^71^. Briefly, approximately 15 g of sieved soil was freeze dried and pulverised in a ball mill. A 500 mg sub-sample was extracted with a chloroform–methanol–citrate buffer mixture (1:2:0.8 v/v), and the phospholipids separated on a silicic acid column. The phospholipids were subjected to a mild-alkali methanolysis and the fatty acid methyl esters extracted into iso-hexane. Fatty acid methyl esters were determined by gas chromatography using a polar capillary column and flame ionisation detection with an internal standard, using the PLFA analysis service of the Macaulay Land Use Institute, Aberdeen, UK. For the replicated Wellesbourne experiments, PLFAs were quantified for 0–10 cm and 10–30 cm samples separately. For the landscape scale study only 0–10 cm samples were quantified.

### Statistical analysis

#### Soil physico-chemical parameters

Replicated land use change experiments: Soil parameters were assessed using a mixed linear model approach, with treatment and depth as fixed effects and block as a random effect. Normality and consistency of variance was determined by examination of residual plots. SOC analyses was done using percentage values rather than carbon stock.

Landscape scale experiment: The effect of set-aside on soil carbon stocks was assessed using a mixed linear model approach. Cropping (set-aside or arable), sampling depth and age of set-aside were added as fixed effects and site as a random effect. The relationship between age and SOC was investigated by fitting age as a polynomial function. There was no improvement in model fit and so the analysis was done as a linear function.

Soil nutrient concentrations and percentage SOC were compared using a mixed linear model with sampling depth included as a fixed affect. Normality and consistency of variance was determined by examination of residual plots. All analyses were done in GenStat version 13 (VSN International Ltd. 2013).

Microbial communities: Differences between set-aside and arable were calculated for individual and total PLFAs using a linear mixed model approach for the replicated experiments and the landscape scale study. Non metric multidimensional scaling (NMDS) was used to compare PLFA profiles in set-aside and arable soil in replicated land use change experiments, across all 17 landscape scale pairs together, and within each individual farm from the landscape scale study separately. NMDS makes few assumptions about distribution of data and is suitable for a wide range of ecological data. Bray-Curtis (Sørensen) resemblance matrices were generated based on presence and abundance of the 50 PLFA measured. The resemblance matrices were plotted in 2-dimensions using NMDS ordination (250 restarts with real data, 250 with randomized (Monte Carlo). Stress (goodness of fit of the plot) was calculated as described by Kruskal^72^, multiplied by 100 to give a percentage value; a stress level of ≤ 5 corresponds to an excellent fit. The significance of differences between treatments after NMDS was assessed using pairwise comparison of all pairs of treatments with a multiple response permutation procedure (MRPP) using Bray-Curtis resemblance. MRPP is a non-parametric procedure that makes no assumptions regarding distributional distances^73^. To determine which PLFAs were contributing most to differences identified by MRPP, similarity percentage analyses (SIMPER) were performed. This method compares average abundances and examines the contribution of each PLFA to similarities within a given group or dissimilarities between groups^74^. All procedures were computed using PCord 5.06 (MjM Software, Gleneden Beach, Oregon, U.S.A.) except SIMPER analysis, performed using PRIMER v6.1.9 software (Primer-E Ltd, Plymouth, UK).

### Data availability

The datasets generated during the current study are available from the corresponding author on reasonable request.

## Electronic supplementary material

Supplementary Information
