# Supplementary material for: Converting highly productive arable cropland in Europe to grassland: –a poor candidate for carbon sequestration
Source: Sci Rep. 2017 Sep 5;7:10493. doi: 10.1038/s41598-017-11083-6 (PMC5585225; doi:10.1038/s41598-017-11083-6)
Supplement: Supplementary file 1 — Supplementary Information [file 41598_2017_11083_MOESM1_ESM.pdf]

Supplementary Information

Converting highly productive arable cropland in Europe to grassland: – a poor candidate for carbon sequestration.

Paul Gosling*[1], Christopher van der Gast[2] and Gary D Bending[3]

Supplementary Table S1. Cropping treatments. Treatments applied in set-aside to arable and arable to set-aside land use conversion experiments at Wellesbourne.

**Arable to set-aside**	**Prior to experiment**	**2007/2008**	**2008/2009**	**2009/2010**
Treatment 1	Arable >30 years	Arable	Arable	Arable
Treatment 2	Arable >30 years	Arable	Set-aside	Set-aside
Treatment 3	Arable >30 years	Arable	Arable	Set-aside
**Set-aside to arable**		**2007/2008**	**2008/2009**	**2009/2010**
Treatment 1	Set-aside 12 years	Set-aside	Set-aside	Set-aside
Treatment 2	Set-aside 12 years	Set-aside	Arable	Arable
Treatment 3	Set-aside 12 years	Set-aside	Set-aside	Arable

Supplementary Table S2. Soil carbon stocks. Soil carbon (t ha^{-1}, 0-30 cm) measured in set aside and cropped land at 14 (17 paired comparisons) sites across England.

Cropping		*Arable*	*Set-aside*
Mean		90.38	88.57
Model Fixed term	*d.f.*	*F statistic*	*P*
Cropping	1,7	0.24	0.643
Age	1,9	0.84	0.607
Cropping.Age	1,9	0.73	0.674

Supplementary Table S3. SOC concentration. Soil carbon(%) measured at different depths in set aside and cropped land at 14 sites (17 paired comparisons) across England.

Depth		*0-10cm*	*10-30cm*
Cropping			
Arable		2.53	2.21
Set-aside		2.87	1.99
Model Fixed term	*d.f.*	*F statistic*	*P*
Cropping	1,47	0.74	0.393
depth	1,47	69.90	<0.001
Cropping.depth	1,47	15.97	<0.001

Supplementary Table S4. SIMPER analysis. Similarity percentages of PLFA contributing more than 5% dissimilarity between set-aside and arable for all site analysis of landscape scale study.

Species	Average Abundance arable	Average Abundance set-aside	Average Dissimilarity	% Contribution	Cumulative %
C18:1w7	4.51	8.26	5.87	14.85	14.85
C18:1w9	2.97	5.02	3.45	8.73	23.58
C16:0	3.12	5.37	3.42	8.65	32.22
phthalate	2.68	4.02	2.84	7.2	39.42
C16:1w7c	2.16	3.69	2.58	6.53	45.94
C19:0cy	1.93	3.14	2.22	5.62	51.57

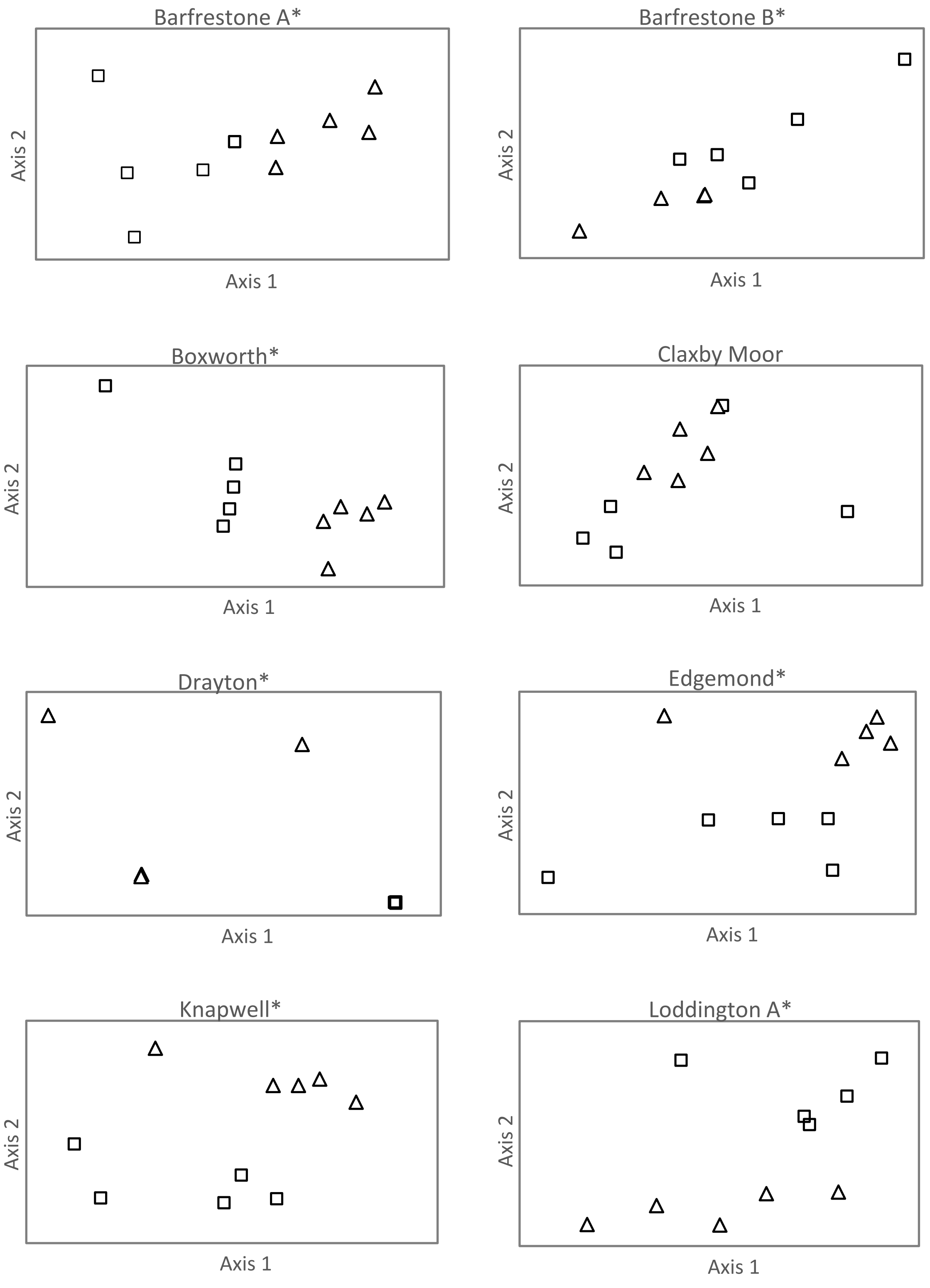
Barfrestone A*
Axis 2
Axis 1
Barfrestone B*
Axis 2
Axis 1
Boxworth*
Axis 2
Axis 1
Claxby Moor
Axis 2
Axis 1
Drayton*
Axis 2
Axis 1
Edgemond*
Axis 2
Axis 1
Knapwell*
Axis 2
Axis 1
Loddington A*
Axis 2
Axis 1

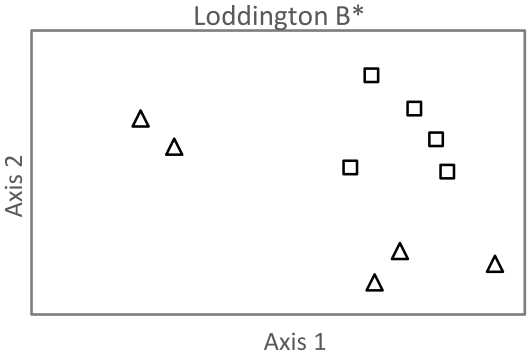
Loddington B*
Axis 2
Axis 1

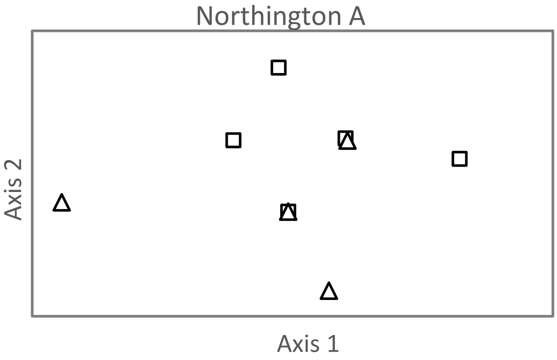
Northington A
Axis 2
Axis 1

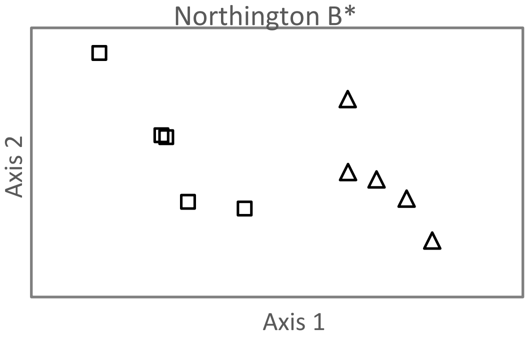
Northington B*
Axis 2
Axis 1

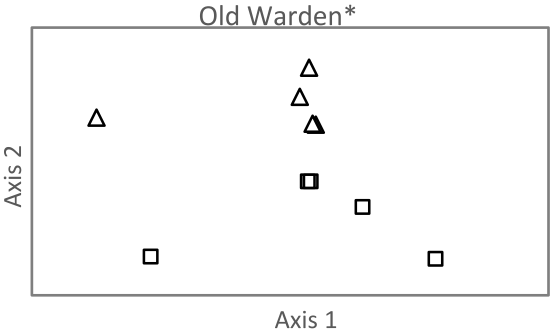
Old Warden*
Axis 2
Axis 1

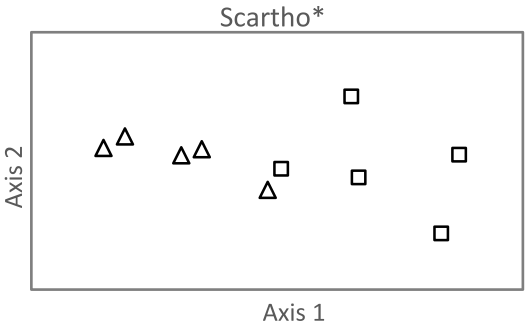
Scartho*
Axis 2
Axis 1

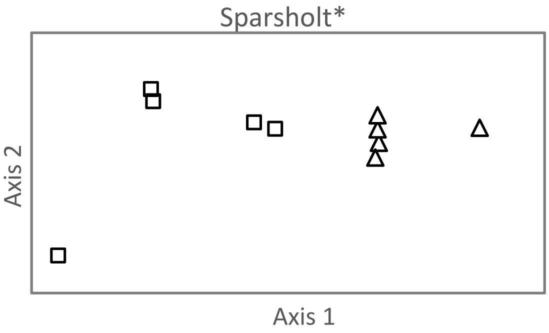
Sparsholt*
Axis 2
Axis 1

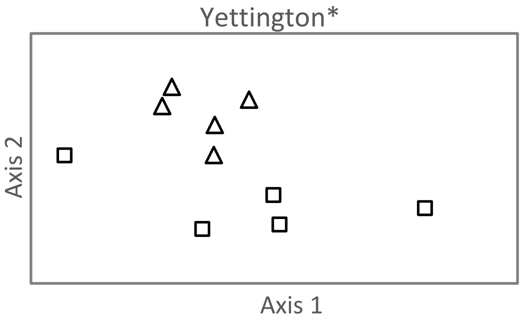
Yettington*
Axis 2
Axis 1

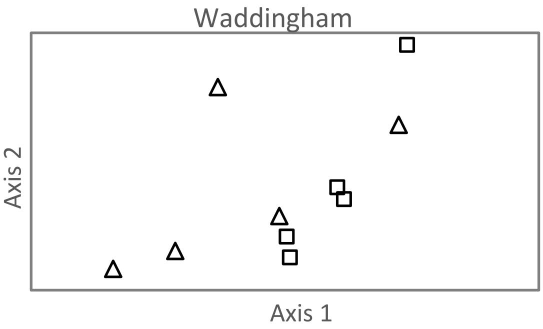
Waddingham
Axis 2
Axis 1

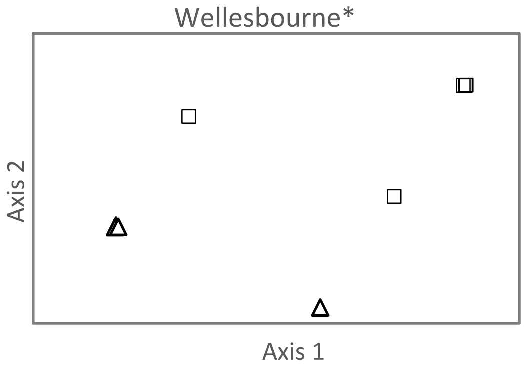

Supplementary Figure S1 NMDS plots of PLFA profiles from soils sampled 0-10 cm for 17 paired arable (□) and set-aside (Δ) farm sites in England. * indicates statistically significant difference between set-aside and arable.
